# MicroRNA-708 regulates *CD38* expression through signaling pathways JNK MAP kinase and PTEN/AKT in human airway smooth muscle cells

**DOI:** 10.1186/s12931-014-0107-0

**Published:** 2014-08-31

**Authors:** Mythili Dileepan, Joseph A Jude, Savita P Rao, Timothy F Walseth, Reynold A Panettieri, Subbaya Subramanian, Mathur S Kannan

**Affiliations:** Department of Veterinary and Biomedical Sciences, College of Veterinary Medicine, 1971 Commonwealth Avenue, St. Paul, MN 55108 USA; University of Pennsylvania Medical Center, Pulmonary, Allergy, & Critical Care Division, Translational Research Laboratories (TRL), 125 South 31st Street, TRL Suite 1200, Philadelphia, PA 19104-3413 USA; Department of Pharmacology, 3-132 BS and BE, 312 Church St SE, Minneapolis, MN 55455 USA; Department of Surgery, University of Minnesota, 11-212 Moos Tower (Mail code: MMC 195), 515 Delaware St, S.E, Minneapolis, MN 55455 USA; Center of Excellence in Environmental Toxicology, University of Pennsylvania Medical Center, Pulmonary, Allergy, & Critical Care Division, Adjunct Professor, Wistar Institute, Translational Research Laboratories (TRL), 125 South 31st Street, TRL Suite 1200, Philadelphia, PA 19104-3413 USA

**Keywords:** MicroRNA, MiR-708, Airway smooth muscle cells, MAP kinase, PI3 kinase, PTEN, AKT, CD38

## Abstract

**Background:**

The cell-surface protein CD38 mediates airway smooth muscle (ASM) contractility by generating cyclic ADP-ribose, a calcium-mobilizing molecule. In human ASM cells, TNF-α augments *CD38* expression transcriptionally by NF-κB and AP-1 activation and involving MAPK and PI3K signaling. *CD38*^*−/−*^ mice develop attenuated airway hyperresponsiveness following allergen or cytokine challenge. The post-transcriptional regulation of *CD38* expression in ASM is relatively less understood. In ASM, microRNAs (miRNAs) regulate inflammation, contractility, and hyperproliferation. The 3’ Untranslated Region (3’UTR) of *CD38* has multiple miRNA binding sites, including a site for miR-708. MiR-708 is known to regulate PI3K/AKT signaling and hyperproliferation of other cell types. We investigated miR-708 expression, its regulation of CD38 expression and the underlying mechanisms involved in such regulation in human ASM cells.

**Methods:**

Growth-arrested human ASM cells from asthmatic and non-asthmatic donors were used. MiRNA and mRNA expression were measured by quantitative real-time PCR. CD38 enzymatic activity was measured by a reverse cyclase assay. Total and phosphorylated MAPKs and PI3K/AKT as well as enzymes that regulate their activation were determined by Western blot analysis of cell lysates following miRNA transfection and TNF-α stimulation. Dual luciferase reporter assays were performed to determine whether miR-708 binds directly to *CD38* 3’UTR to alter gene expression.

**Results:**

Using target prediction algorithms, we identified several miRNAs with potential *CD38* 3’UTR target sites and determined miR-708 as a potential candidate for regulation of *CD38* expression based on its expression and regulation by TNF-α. TNF-α caused a decrease in miR-708 expression in cells from non-asthmatics while it increased its expression in cells from asthmatics. Dual luciferase reporter assays in NIH-3 T3 cells revealed regulation of expression by direct binding of miR-708 to *CD38* 3’UTR. In ASM cells, miR-708 decreased *CD38* expression by decreasing phosphorylation of JNK MAPK and AKT. These effects were associated with increased expression of MKP-1, a MAP kinase phosphatase and PTEN, a phosphatase that terminates PI3 kinase signaling.

**Conclusions:**

In human ASM cells, TNF-α-induced *CD38* expression is regulated by miR-708 directly binding to 3’UTR and indirectly by regulating JNK MAPK and PI3K/AKT signaling and has the potential to control airway inflammation, ASM contractility and proliferation.

## Background

The cell-surface protein CD38 mediates calcium signaling in airway smooth muscle (ASM) and innate immune responses [1]. ADP-ribosyl cyclase activity of CD38 converts β-nicotinamide adenine dinucleotide (β-NAD) to cyclic adenosine diphosphoribose (cADPR) and adenosine diphosphoribose (ADPR) [2]. In previous investigations in ASM, we showed that cADPR mediates calcium release from the sarcoplasmic reticulum and promotes contractility [3,4]. Furthermore, *CD38*^*−/−*^ mice exhibit attenuated methacholine responsiveness and airway hyperresponsiveness (AHR) following allergen sensitization and challenge as well as after intranasal IL-13 challenge [5–7]. ASM cells obtained from *CD38*^*−/−*^ mice exhibit attenuated intracellular calcium responses to contractile agonists relative to cells obtained from wild-type mice [6]. These observations indicate a prominent role of *CD38* in AHR, a cardinal feature of asthma in humans.

The transcriptional regulation of *CD38* in ASM involves the transcription factors NF-κB and AP-1 and signal transduction mechanisms involving activation of MAP kinases and PI3 kinase [8,9]. *CD38* is ubiquitously expressed in many cell types in addition to ASM cells and its expression is augmented by inflammatory and Th2 cytokines [4,10–16]. While the transcriptional regulation of *CD38* expression has been thoroughly investigated in mammalian cells, there is paucity of information regarding post-transcriptional regulation of its expression. In this regard, we recently reported evidence for such regulation involving the microRNA (miRNA) miR-140-3p [17]. In human ASM cells, human recombinant TNF-α-(*rh*-TNF-α)-induced *CD38* expression is attenuated by miR-140-3p through both direct binding to the 3’ Untranslated Region (3’UTR) of *CD38* as well as indirect mechanisms involving activation of p38 MAP kinase and the transcription factor NF-κB. *CD38* 3’UTR is ~481b long and has multiple miRNA binding sites, including a site for miR-708. Prior studies have revealed a prominent regulatory role of miR-708 in the expression of phosphatase and tensin homolog (*PTEN*), which in turn regulates PI3 kinase signaling through activation of AKT [18]. In other cell types, PI3 kinase/AKT signaling regulates expression of target genes by activating the proinflammatory transcription factor NF-κB [19]. An effect of miR-708 on *PTEN* expression is expected to profoundly affect cytokine-induced *CD38* expression in ASM cells by modulating PI3 kinase signaling. In ASM cells obtained from asthmatics, *rh*-TNF-α induces significantly greater *CD38* expression compared to its expression in cells from non-asthmatics [20]. In this study, we investigated the expression of miR-708, its potential additive role with miR-140-3p in the regulation of *CD38* expression and the underlying mechanisms involved in such regulation in human ASM cells. We also examined miR-708 expression and its effects on *CD38* expression in ASM cells obtained from asthmatics to determine whether the augmented cytokine-mediated *CD38* expression stems from altered regulation through miR-708.

## Methods

### Reagents

DMEM was from GIBCO-BRL (Grand Island, NY); *rh*-TNF-α was from R&D Systems (Minneapolis, MN); TRIzol, SuperScript III reverse transcriptase, NCode miRNA first-strand synthesis kit, Platinum SYBR Green quantitative PCR (qPCR) mix, Opti-MEM® reduced serum medium and Lipofectamine® RNAiMax transfection reagent were from Invitrogen Life Technologies (Carlsbad, CA); Brilliant lll Ultra-Fast SYBR Green q-PCR Master Mix from Agilent Technologies, Inc (Santa Clara CA); Fugene HD transfection reagent was from Roche Diagnostics (Indianapolis, IN); QuikChange Lightning Multi Site-Directed mutagenesis kit was from Agilent Technologies, Inc (Santa Clara, CA); control oligo (scrambled sequence mimic), miR-708 mimic (mature miR-708 sequence: 5’-AAGGAGCUUACAAUCUAGCUGGG-3’), and antagomir oligonucleotides were from Dharmacon (Lafayette, CO); Dual Luciferase Reporter Assay System was from Promega (San Luis Obispo, CA); NIH-3 T3 cells were from ATCC (#CRL-1658, Manassas, VA); chemiluminescent substrate for horseradish peroxidase (HRP) was from Millipore (Billerica, MA); rabbit primary antibodies against major MAPK family, PTEN, AKT2, AKT and β-actin as well as anti-rabbit secondary antibody were from Cell Signaling Technology (Danvers, MA); mouse primary antibodies for MKP-1, α-actin and goat-anti mouse antibodies were from Santa Cruz Biotechnology (Dallas, TX). Tris-base, glucose, HEPES and other chemicals were from Sigma Chemical Co. (St. Louis, MO) unless otherwise mentioned.

### Culture and transfection of HASM cells

Isolation, culture and maintenance of HASM cells was carried out as described in our previous publications [4,17,21]. Briefly, HASM cells obtained from unidentified healthy donors or from fatal asthmatics were maintained in culture and used until the smooth muscle phenotype is sustained (2–5 passages). DMEM supplemented with 10% FBS, 100 U/ml penicillin, 0.1 mg/ml streptomycin, and 0.25 g/ml amphotericin B was used to grow the HASM cells up to 80% confluency. Rh-TNF-α (10 ng/ml) was used to induce the expression of CD38 in HASM cells while control cells were treated with 0.1% BSA in PBS. Since human cells used in this study were obtained from unidentified donors, it is considered Exempt under National Institutes of Health guidelines. Drs. Panettieri and Kannan have Institutional Review Board approval for the use of these cells in the study.

Transient transfection of primary HASM cells with miR-708 mimic, antagomir or scrambled sequence mimic at 10–100 nM was carried out in the presence of Lipofectamine® RNAiMax transfection reagent. Reduced serum Opti-MEM® medium was used as a base to prepare the transfection complex containing Lipofectamine and the oligonucleotides. After 20 min of incubation at room temperature the transfection complex was gently dropped on to the cell suspension (1.5-2.0 × 10^5^ cells/well in 24 well plates or 2.5-3.0 × 10^5^ cells/well in 6 well plates) which was seeded few minutes (<3 min) prior to the addition of transfection complex. Transfected cells were incubated at 37°C for 24 h, growth arrested for 24 h, exposed to *rh-*TNF-α for another 24 h before isolation of total RNA or total protein.

### Total RNA isolation

PureLink and *mir*Vana RNA isolation kits (Ambion Life Technologies, Carlsbad, CA) were used to isolate total large RNA and small RNA, respectively, according to the manufacturer’s instruction.

### cDNA synthesis and quantitative q-PCR

NCode™ miRNA First-Strand-cDNA synthesis kit was used to synthesize cDNA followed by q-PCR for miRNAs using the Platinum q-PCR Kit according to the manufacturer’s instruction. Briefly, expression of miR-708 at constitutive levels and after induction with *rh-*TNF-α was measured by q-PCR after poly-adenylation. Whole miR-708 sequence was employed as a specific forward primer and universal q-PCR primer was used as a reverse primer. Mammalian small nuclear RNA U6, a spliceosomal RNA, served as a control housekeeping gene. To measure changes in the expression of *CD38* [8,20] and *JNK* at transcript levels (following miR-708 mimic or scrambled sequence mimic transfection), q-PCR was performed using Brilliant SYBR Green Master Mix. Primers for JNK were selected using Primer-BLAST (http://www.ncbi.nlm.nih.gov/tools/primer-blast/) and performed in the Stratagene Mx3000p q-PCR system, under the following conditions: 1 cycle of 95°C for 5-min segment, 40 cycles of 95°C for 30 s, 59°C for 30 s, 72°C for 45 s, and a final 1 cycle of 95°C for 1 min, 59°C for 30s and 95°C for 30s (JNK forward primer 5’-CACCACCAAAGATCCCTGACA-3’; JNK reverse primer 5’-CTGTGCTAAAGGAGAGGGCT-3’). Expression level of *cyclophilin* was used to normalize the expressions of *CD38* and *JNK*.

### mRNA stability

HASM cells were transfected with miR-708 mimic or scrambled sequence mimic. After 24 h, transfection medium was replaced with growth-arrest medium for 24 h. Cells were then exposed to *rh-*TNF-α for 12 h in fresh growth arrest medium which was replaced with fresh growth arrest medium containing actinomycin D (5 μg/ml) to inhibit transcription. At different time points (0, 6, 12 and 24 h), cells were collected for isolation of total RNA to evaluate the rate of decay of CD38 transcript by q-PCR.

### Western blot

Twenty four hours after transfection of ASM cells with miRNA, cell growth was arrested for 24 h (with serum-free media containing transferrin and insulin) followed by treatment with *rh-*TNF-α for an additional 24 h. Cells were collected in the lysis buffer (50 mM Tris, 100 mM NaCl, 50 mM NaF, 40 mM β-glycerol phosphate, 2 mM EDTA, 0.2 mM Na_3_VO_4_, 1% Triton X-100, and protease inhibitor cocktail, pH 7.4), lysed by sonication and total protein concentration in the lysates estimated by Bradford assay (Bio-Rad Laboratories, Inc, Hercules, CA). Equal amounts of total protein (5–15 μg) were separated by SDS-PAGE (4-20% gradient gels), transferred onto a PVDF membrane and blocked overnight in 5% skim milk in PBS containing 0.05% Tween 20 (PBST) at 4°C. Incubation of membranes with primary antibodies was carried out in 1% skim milk in PBST at 4°C overnight. After three 5-minute washes with PBST, membranes were incubated with HRP-conjugated secondary antibodies in 1% milk for 1 h at room temperature. Primary rabbit antibodies against all major MAPKs, β-actin, PTEN, Pan AKT and AKT2 were used at a dilution of 1:1000 except antibodies to detect p38 and phospho-p38 which were used at a dilution of 1:500. Primary mouse antibodies (smooth muscle-specific α-actin and MKP-1) were used at a dilution of 1:700. Anti-rabbit secondary antibodies were used at a dilution of 1:5000, while goat anti-mouse secondary antibodies were used at a dilution of 1:4000. To determine the activation of MAPKs and AKT following miR-708 transfection, cells were collected at 20 min or 2 h respectively after treatment with *rh-*TNF-α and lysed. After electrophoresis, same membranes were probed for phosphorylated and total protein expression, stripping membranes (Restore Plus Western Blot Stripping Buffer [Thermo Fisher Scientific Co., Pittsburgh], 30 min) between blots. Intensity of protein bands was measured using ImageJ image analysis software [22] and the level of expression of phosphorylated protein relative to total protein was considered as a measure of activation. In the case of MKP-1, PTEN and total MAP kinase protein expression, cells were collected 24 h after stimulation with *rh-*TNF-α and changes in expression level relative to loading control (smooth muscle specific α-actin or β- actin) were measured.

### Reverse ADP-ribosyl cyclase assay

The enzymatic function of CD38 was quantified by reverse cyclase assay as described in our earlier publication [20]. Briefly, equal amount of (5 -15 μg) total proteins in human ASM (HASM) cell lysates were incubated at 37°C for 1 h with cADPR (0.45 mM) in the presence or absence of nicotinamide (10 mM). The enzymatic reaction was terminated by adding 25 μl of 1 M HCl, and contents were vacuum filtered (0.45 μm, Immobilon, Millipore), neutralized (15 μl of 2 M Tris-base), and incubated at room temperature with a mixture of the following reagents: rezasurin (2 μM), ethanol, flavin mononucleotide (4 μM), alcohol dehydrogenase (40 μg/ml) and diaphorase (0.04 U/ml) in phosphate buffer (Na_2_HPO_4_/NaH_2_PO_4_). FLUO star Galaxy fluorometer was used to quantify the rate of fluorescence emitted at 590 nm which is proportionate to the amount of NAD generated by the CD38 present in 5–15 μg of total protein.

### Mutation of miR-708 target site and dual luciferase reporter assay

Location of miR-708 target site at the 3’UTR of CD38 transcript is 21 bases away from the stop codon. Mutation of four bases (ctCgCCg) at the target site (AGCTCCT) specific for miR-708 was achieved using the QuikChange Lightning Multi Site-Directed mutagenesis kit. The primer sequence designed for the mutation consists of target sequence and 14 bases flanking the target sequence (5’-GCTGTGGTTGTTTT***CTCGCCG***TGACTCCTTGTGGT-3’). XL10-GOLD Ultra competent *E. coli* was used to expand the wild-type and mutant plasmids. NIH-3 T3 cells seeded in 24 well plates (1.5-2.0 × 10^5^ cells/well) were co-transfected with miR-708 mimic or scrambled sequence mimic (50 nM), wild-type (or mutated) firefly Luc-CD38-3’UTR-reporter plasmid (200 ng/well) and Renilla luciferase plasmid (control) (30 ng/well), in culture medium (Opti-MEM® reduced serum medium; 100 μl), facilitated by Fugene HD transfection reagent (1 μl/well). After 24 h, cells were collected in lysis buffer and luciferase activity in triplicate samples for each condition was measured with a luminometer according to the manufacturer’s recommendation (Synergy 2 microplate reader, BioTek, Winooski, VT). Renilla luciferase activity was used to normalize the firefly luciferase activity.

### Statistical analysis

HASM cells obtained from 3–8 different donors were used. Values represent means ± SEM. Data were analyzed for statistical significance by Student’s *t*-test or one-way ANOVA (depending on the number of experimental groups analyzed) using GraphPad Prism 6 Software. Differences were considered significant at p < 0.05.

## Results

### Differential expression of miR-708 in HASM cells

**Figure 1:**
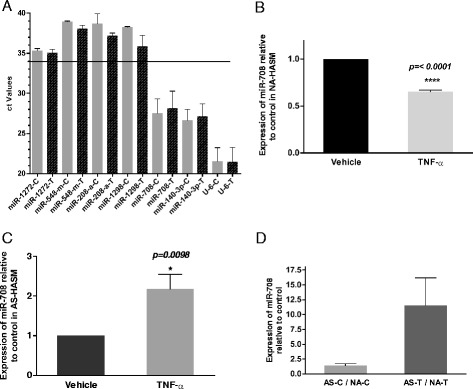
**Expression of miR-708 in** ***rh-*** **TNF-α-treated ASM cells.** **A** *:* Analysis of miRNAs that are predicted to target *CD38* by q-PCR. Expression levels of these miRNAs in HASM cells with and without TNF-α treatment were normalized with small nuclear RNA U6. miRNAs with ct values above and below 34 in HASM cells are indicated by the horizontal line (n = 3–4 donors). **B** and **C** *:* miR-708 expression in HASM cells from non-asthmatics (NA-HASM) and asthmatics (AS-HASM), respectively. *Rh-*TNF-α caused a significant reduction in miR-708 expression in growth-arrested NA-HASM cells compared with cells treated with vehicle (n = 8 donors), while it caused a significant increase in its expression in AS-HASM cells (n = 6 donors). **D** *:* miR-708 expression in AS-HASM cells is higher than in NA-HASM cells both under vehicle and TNF-α-treated conditions (3–5 donors/group). Values are shown as mean ± SEM. C-vehicle-treated cells (0.1% BSA); T- *rh*-TNF-α (10 ng/ml) treated cells.

In order to investigate the potential regulation of *CD38* expression by multiple miRNAs, we first analyzed the *CD38* 3’UTR for miRNA binding sites using target prediction algorithms. MiRNAs miR-1272, miR-548, miR-208a, miR-1298, miR-708 and miR140-3p were predicted to bind to the *CD38* 3’UTR with high context score. Expression levels of these miRNAs in HASM cells were evaluated by q-PCR. As co-expression of miRNA(s) along with its target gene transcript is required for regulation, using a cycle threshold (ct) cut-off, miRNAs with ct values above 34 in HASM cells were filtered out (Figure 1A). Other miRNAs (miR-708 and miR140-3p) which showed ct values ranging from 20–30 (Figure 1A) were selected for further studies.

To evaluate the expression of miR-708 in HASM cells obtained from non-asthmatic and asthmatic donors in the presence of *rh-*TNF-α, q-PCR was performed. In non-asthmatic HASM (NA-HASM) cells, *rh-*TNF-α caused a significant (p < 0.0001) reduction in the expression of miR-708 (Figure 1B) compared to expression in unstimulated (vehicle-treated) cells. In contrast, *rh-*TNF-α exposure significantly increased the expression of miR-708 (p = 0.0098) in asthmatic-HASM (AS-HASM) cells (Figure 1C). Further, expression of miR-708 in AS-HASM cells was found to be higher in vehicle treated (~2-fold) and *rh-*TNF-α treated (>10-fold) cells when compared to NA-HASM cells (Figure 1D).

### miR-708 inhibits *CD38* expression and its enzymatic activity in HASM cells

**Figure 2:**
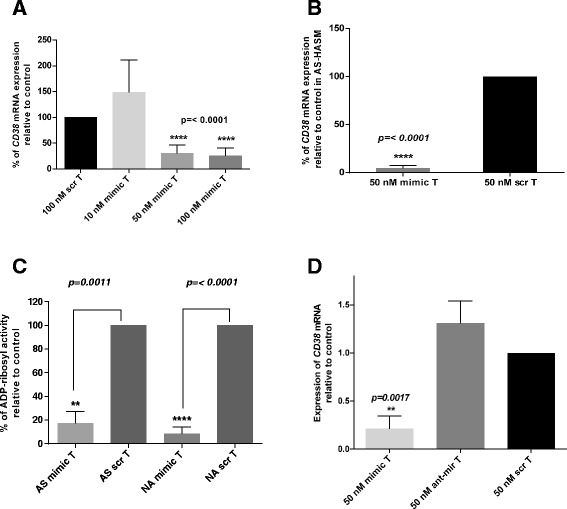
**MiR-708 inhibits** ***CD38*** **expression in HASM cells.** **A**: NA-HASM cells were transfected with different concentrations of miR-708 mimic (mimic) or scrambled sequence mimic (scr), followed by exposure to 10 ng/ml *rh-*TNF-α. CD38 transcript levels were measured by q-PCR. Results are shown as % CD38 mRNA relative to scrambled sequence mimic (scr) transfection. Significant inhibition of *CD38* expression was observed following transfection of miR-708 mimic at concentrations ≥ 50 nM (n = 3–5 donors). **B**: CD38 transcript levels following transfection with miR-708 mimic (50 nM) or scrambled sequence mimic in AS-HASM cells. Note significant attenuation of *CD38* expression by miR-708 mimic transfection (n = 3 donors); **C**: ADP-ribosyl cyclase activity in AS-HASM (AS) and NA-HASM (NA) cells following transfection with miR-708 mimic or scrambled sequence mimic and exposure to *rh-*TNF-α (MT and ST, respectively, (n = 3 donors). Note significant attenuation of enzyme activity in cells from AS and NA after transfection with miR-708 mimic. **D**: CD38 transcript levels in NA-HASM cells following transfection with miR-708 mimic (50 nM), scrambled sequence mimic or antagomir for the miRNA (ant-mir) (n = 3 donors). Note the lack of inhibition of *CD38* expression in cells transfected with ant-mir. Data represents mean ± SEM.

To examine whether miR-708 alters the expression level of *CD38*, NA-HASM cells were transiently transfected with different concentrations of miR-708 mimic. Over-expression of miR-708 mimic at 50 nM and 100 nM concentrations significantly decreased CD38 transcript level (Figure 2A). This finding was further confirmed in AS-HASM where miR-708 mimic at 50 nM significantly inhibited CD38 transcript level relative to the scrambled sequence mimic (Figure 2B). Further, transfection of AS-HASM cells and NA-HASM cells with miR-708 at a concentration of 50 nM decreased the enzymatic activity of CD38 measured by ADP-ribosyl cyclase assay (Figure 2C). To establish the specificity of the inhibitory effect of endogenous miR-708 on *CD38* expression, NA-HASM cells were transfected with miR-708 mimic, scrambled sequence mimic or the antagomir of miR-708 mimic. *Rh-*TNF-α-induced *CD38* expression following miR-708-antagomir transfection was similar to the expression in cells transfected with scrambled sequence mimic (Figure 2D).

**Figure 3:**
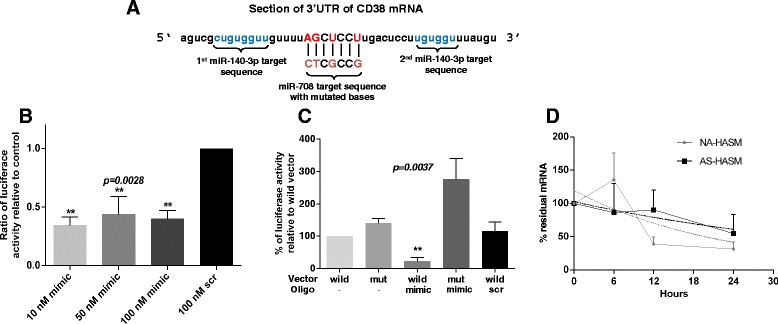
**Interaction of miR-708 with the 3’UTR of CD38.** **A**: Predicted binding sites for miR-708 and miR-140-3p in 3’UTR of CD38. Shown are the mutated bases in the miR-708 target site of the 3’UTR of *CD38*. **B**: Relative luciferase activity in NIH-3 T3 cells co-transfected with reporter plasmid and different concentrations of miR-708 mimic (mimic) or scrambled sequence mimic (scr) at the highest concentration (n = 3, experiments in triplicate). Note significant reduction in luciferase activity following transfection with miR-708 mimic at all concentrations. **C**: Relative luciferase activity in cells co-transfected with reporter plasmid containing either wild-type (wild) or mutant (mut) *CD38* 3’UTR followed by transfection with 50 nM of miR-708 mimic (mimic) or scrambled sequence mimic (scr) (n = 3, experiments in triplicate). Note no inhibition of luciferase activity in cells co-transfected with mutant *CD38* 3’UTR. **D**: CD38 mRNA stability measured in growth-arrested HASM cells. Cells were transfected with 50 nM miR-708 and exposed to *rh-*TNF-α in the presence of actinomycin D. At time points indicated, CD38 transcript levels were quantified by q-PCR (n = 2–5 donors). Graph shows plots of non-linear regression of mRNA decay over time for NA-HASM and AS-HASM cells, with the dotted lines indicating the best fit. Values are means ± SEM for results shown.

**Figure 4:**
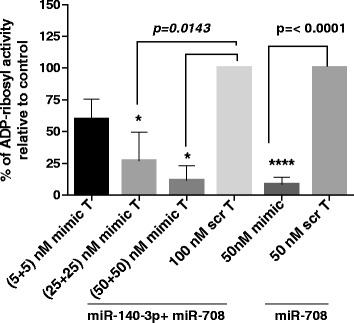
**Effect of miR-708 in combination with miR-140-3p on ADP-ribosyl cyclase activity in NA-HASM cells.** Cells (n = 3 donors) were co-transfected with equimolar concentrations of miR-708 and miR-140-3p, miR-708 mimic alone or the corresponding control scrambled sequence mimic at the indicated concentrations followed by exposure to 10 ng/ml *rh-*TNF-α (T) and measurement of ADP-ribosyl cyclase activity. Data represents mean ± SEM. Note that no additive effect was observed in cells transfected with equimolar concentrations of miR-708 and miR-140-3p relative to miR-708 alone.

Our previous studies have identified a role for miR-140-3p in the regulation of cytokine-induced CD38 gene expression and enzyme activity independent of miR-708 [17]. Since the target sites of these miRNAs are closely situated in the 3’UTR of CD38 transcript (see Figure 3A), we examined whether transfection of HASM cells with both miR-140-3p and miR-708 would amplify the inhibitory effect on enzymatic activity of CD38. Co-transfection of HASM cell with miR-140-3p and miR-708 at 50 nM and 100 nM (equimolar concentrations) significantly inhibited CD38 enzymatic activity relative to cells transfected with scrambled sequence mimic. However there was no evidence of an additive effect (Figure 4).

### MiR-708 directly binds to 3’UTR of *CD38*

MiRNAs can inhibit gene expression directly by binding to the target gene at the 3’UTR or indirectly by inhibiting multiple components in the signaling pathway [23,24]. We first examined whether miR-708 regulates the expression of CD38 by directly binding to its 3’UTR by performing dual luciferase reporter assays in a heterologous cell system (NIH-3 T3 cells). Four bases in the target sequence on the CD38 3’UTR were mutated to establish specificity of miR-708 target binding (Figure 3A). A significant reduction in relative luciferase activity was noted when miR-708 mimic and reporter plasmid were co-transfected compared to co-transfection with a scrambled sequence mimic (Figure 3B). Mutation of four bases in the target sequence on the *CD38* 3’UTR reversed the inhibitory effect of miR-708 on luciferase activity confirming the specificity of its target binding at the 3’UTR of *CD38* (Figure 3C). To examine whether this binding leads to mRNA degradation, CD38 mRNA stability was measured in HASM cells transfected with miR-708 mimic or scrambled sequence mimic and exposed to *rh*-TNF-α followed by actinomycin D for transcriptional arrest. For analysis of mRNA stability, the amount of CD38 mRNA remaining at each time point up to 24 h relative to 0 h was determined by q-PCR using the 2^-ΔΔCt^ calculation method and plotting against time. Nonlinear regression analysis of biological replicate samples was fitted using one-phase decay kinetics (GraphPad Prism 6). In NA- and AS-ASM cells transfected with miR-708 mimic or the scrambled sequence mimic, there was comparable decay in CD38 mRNA content, with half-life ranging from 16–32 h (Figure 3D).

### Transcriptional regulation of *CD38* expression by miR-708

**Figure 5:**
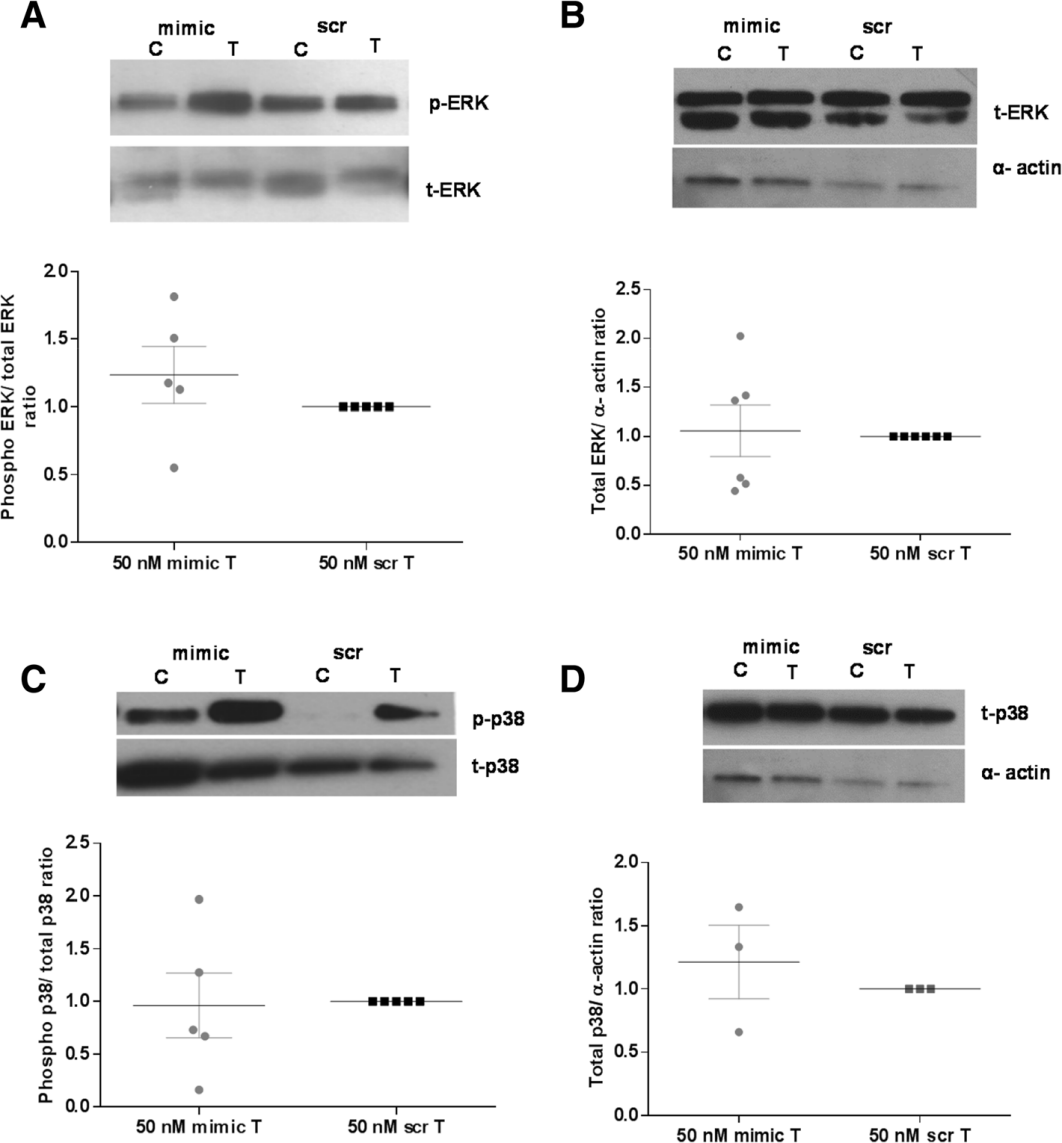
**Effect of miR-708 transfection on ERK and p38 MAP kinase activation and expression in NA-HASM cells.** Western blot analysis of cell lysates following transfection with 50 nM miR-708 mimic (mimic) or scrambled mimic (scr) and treatment with *rh-*TNF-α for 20 min (for total and phosphorylated levels) or 24 h (for total expression levels) were performed with antibodies against phosphorylated and total ERK **(A and B)** as well as p38 **(C and D)**, respectively. Note no significant change in expression of total or phosphorylated ERK (n = 5–6 donors) and p38 (n = 3–5 donors) following miR-708 mimic transfection. Each Western blot is a representative blot from a single donor. Values are means ± SEM for results shown. C-vehicle-treated cells (0.1%BSA); T- *rh-*TNF-α (10 ng/ml) treated cells.

**Figure 6:**
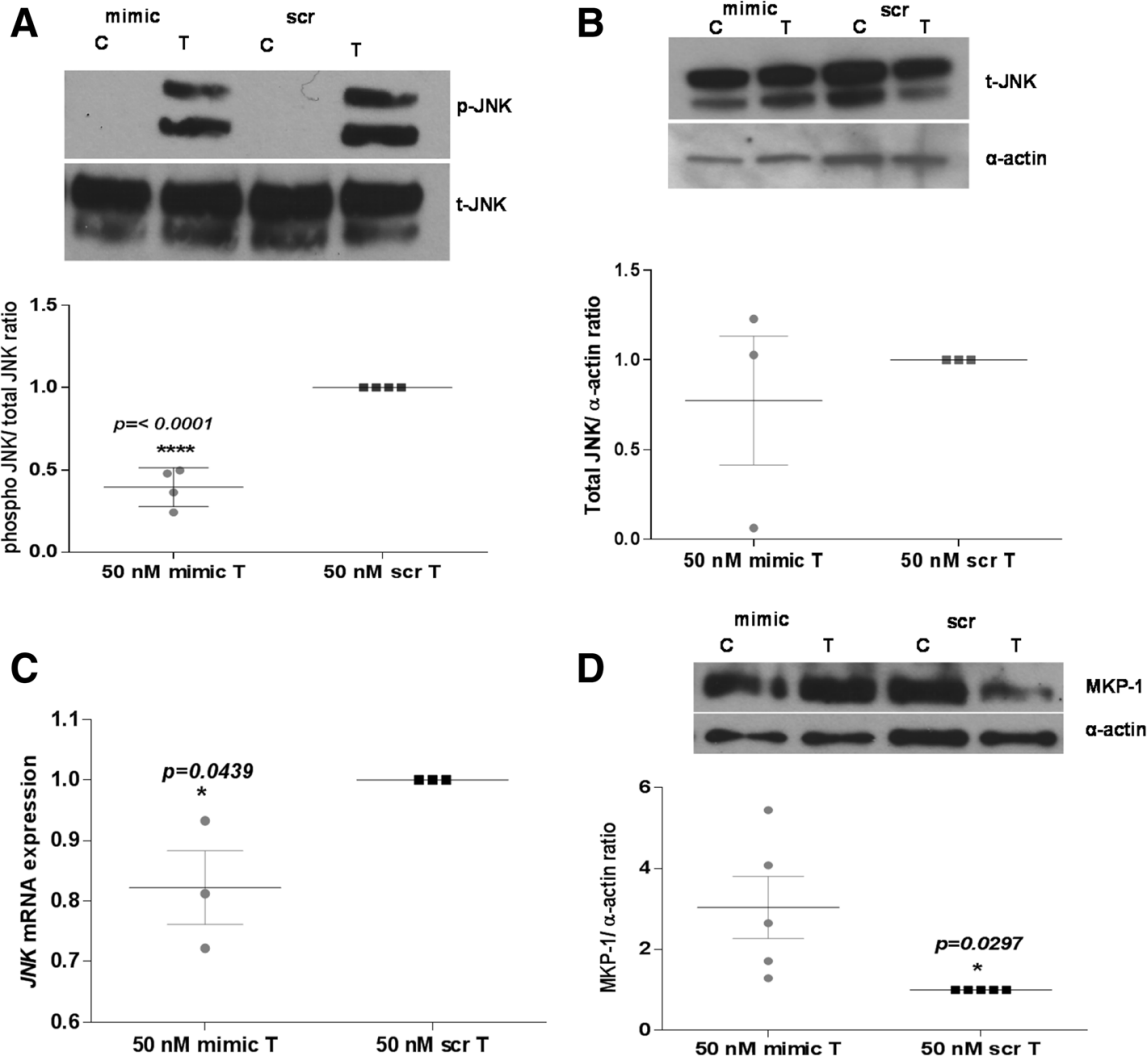
**Effect of miR-708 transfection on JNK MAP kinase signaling in NA-HASM cells.** **A** *and* **B**: Western blot analysis of cell lysates following transfection with 50 nM miR-708 mimic (mimic) or scrambled sequence mimic (scr) was performed as described in Figure 5 to detect JNK MAP kinase after *rh-*TNF-α treatment for 20 min (total and phosphorylated levels) (n = 4 donors) as well as total JNK expression after *rh-*TNF-α treatment for 24 h (n = 3 donors). Note significant down-regulation of JNK MAP kinase phosphorylation but no change in total JNK protein expression in mimic-transfected cells compared to cells transfected with scr. **C**: Total RNA extracted from cells transfected with mimic or scr and treated with *rh-*TNF-α (24 h) was analyzed for JNK mRNA expression by q-PCR (n = 3 donors). Note significant down-regulation of JNK expression by mimic. **D**: MKP-1 expression in cells transfected with mimic or scr and treated with *rh-*TNF-α for 24 h was examined by Western blot analysis (n = 5 donors). Note significant up-regulation of MKP-1 expression in mimic-transfected cells compared to scr. Each Western blot is a representative blot from a single donor. Values are means + SEM for results shown. C-vehicle-treated cells (0.1% BSA); T-TNF-α (10 ng/ml) treated cells.

In prior studies, we reported that cytokine-induced changes in *CD38* expression in HASM cells involve activation of MAP kinases and PI3 kinases. Among the MAP kinases, p38 and JNK MAP kinases were found to regulate expression transcriptionally while ERK MAP kinase was involved in regulation post-transcriptionally through transcript stability [8]. In this study, we examined the effect of miR-708 transfection following TNF-α exposure (20 min) on levels of total MAP kinase protein as well as phosphorylated (activated) protein in order to determine whether decreased MAP kinase activation is an underlying mechanism in the regulation of *CD38* expression. Cells were transfected with miR-708 mimic or its scrambled sequence mimic, exposed to *rh-*TNF-α for 24 h and expression levels of the MAP kinases determined by Western blot analysis of cell lysates. Transfection of HASM cells with miR-708 mimic had little effect on levels of phosphorylated or total ERK and p38 (Figure 5). A significant reduction, however, was noted in the level of phosphorylated JNK MAP kinase in growth-arrested, TNF-α exposed (20 min) cells following miR-708 transfection (Figure 6A). Further, although total JNK MAP kinase expression remained unaltered in these cells (Figure 6B), there was a significant reduction in JNK mRNA expression (Figure 6C). We next examined whether reduced JNK phosphorylation might be a consequence of increased expression of a phosphatase. Expression of a MAP kinase phosphatase, MKP-1, was measured by Western blot in miR-708-transfected cells following *rh-*TNF-α exposure. MKP-1 expression was significantly higher in cells transfected with miR-708 mimic compared to cells transfected with the scrambled sequence mimic (Figure 6D). These results suggest that decreased JNK MAP kinase phosphorylation caused by increased expression of MKP-1 may be involved in miR-708 regulation of *CD38* expression in HASM cells.

**Figure 7:**
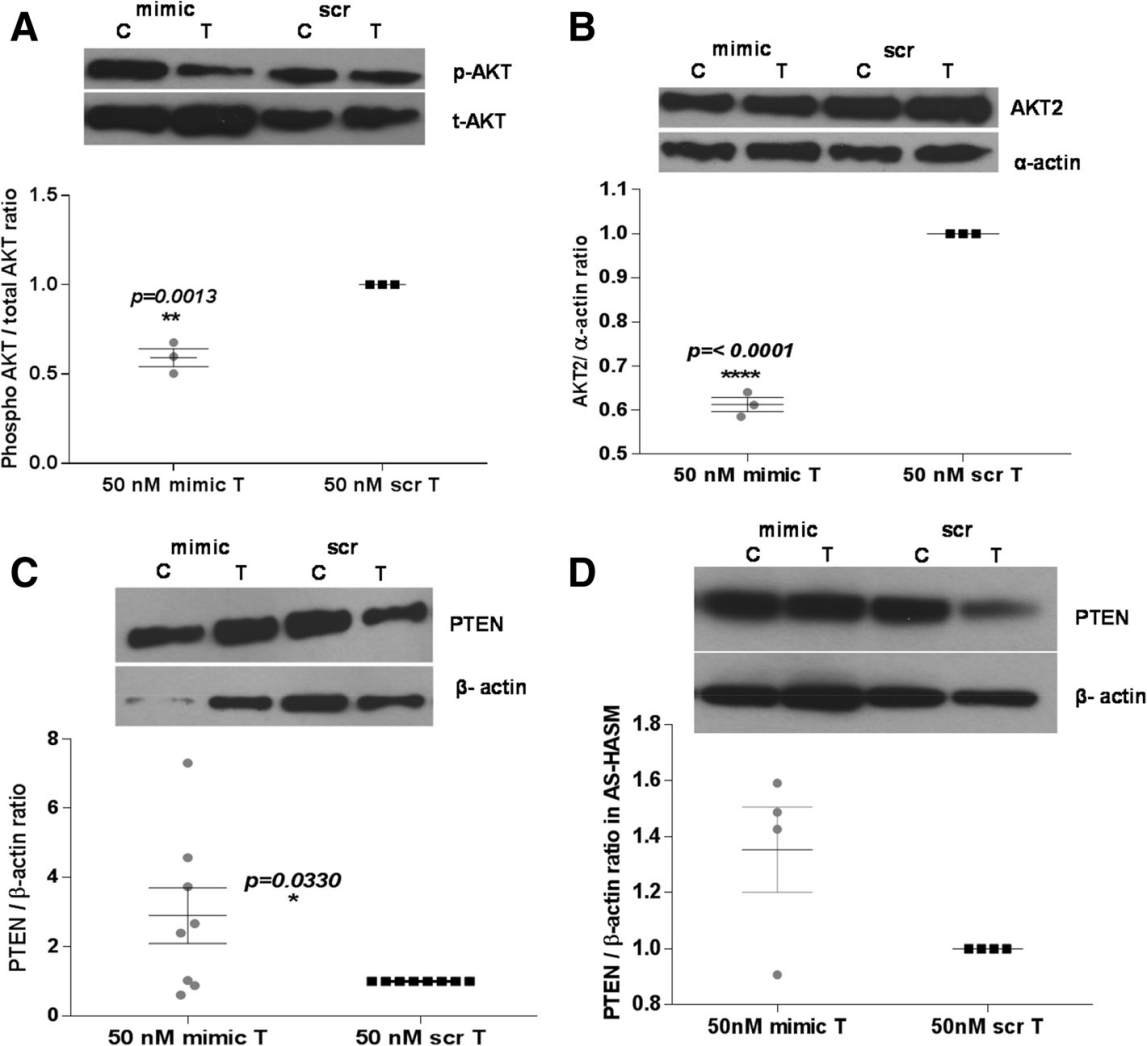
**Effect of miR-708 transfection on PTEN/AKT signaling.** **A**, **B** and **C**: NA-HASM cells were transfected with 50 nM miR-708 mimic (mimic) or scrambled sequence mimic (scr) and treated with *rh-*TNF-α for 2 h to measure total and phosphorylated AKT (n = 3 donors) or 24 h to measure AKT2 (n = 3 donors) and PTEN expression (n = 8 donors) by Western blot analysis of cell lysates. Note significant down-regulation of AKT phosphorylation and AKT2 expression and up-regulation of PTEN expression. **D**: PTEN expression in AS-HASM cells was higher (not significant) in samples from 3 of 4 donors. Each Western blot is a representative blot from a single donor. Values are means ± SEM for results shown. C-vehicle-treated cells (0.1% BSA); T-TNF-α (10 ng/ml) treated cells.

In a prior study, we reported that regulation of *CD38* expression in HASM cells involves activation of PI3 kinases [9]. Furthermore, PI3 kinase signaling is regulated by other miRNAs, including miR-708 [25]. Therefore, we measured levels of phosphorylated AKT, a molecule in the PI3 kinase signaling cascade, as well as levels of PTEN, a phosphatase that terminates PI3 kinase signaling. In cells transfected with miR-708 mimic, the level of phosphorylated AKT was significantly lower relative to cells transfected with the scrambled sequence mimic (Figure 7A). Further, a significant reduction was also noted in the expression of AKT2 (Figure 7B), an isoform of AKT that has a 3’UTR binding site for miR-708 [25]. In consistence with an overall attenuation of PI3 kinase signaling in miR-708-mimic-transfected cells, there was a significant increase in PTEN expression compared to expression in cells transfected with the scrambled sequence mimic (Figure 7C). A similar increase in PTEN expression was observed in AS-HASM cells following miR-708 mimic transfection compared to cells transfected with the scrambled sequence mimic (Figure 7D).

## Discussion

The cell-surface protein CD38 is known to contribute to calcium regulation and contractility of ASM [4]. In prior investigations, we and others have shown that in ASM cells calcium release through ryanodine receptor channels in the sarcoplasmic reticulum during agonist stimulation involves cyclic ADP-ribose (cADPR) [4,26,27]. This involves the enzymatic activity of CD38, i.e., ADP-ribosyl cyclase, which converts β-NAD to cADPR [2,28]. Furthermore, exposure of ASM cells to inflammatory cytokines results in significantly augmented *CD38* expression and cADPR-mediated intracellular calcium release [4]. These observations strongly implicate the CD38/cADPR pathway of calcium signaling in ASM hyperresponsiveness, a hallmark of asthma. *CD38* deficient mice are also hyporesponsive to inhaled methacholine following sensitization and challenge with allergen [7] as well as following challenge with inhaled inflammatory cytokines [5,29]. These mice develop a robust airway inflammatory response following allergen or cytokine challenge, although ASM obtained from these mice exhibit significantly attenuated contractile responses to relevant airway spasmogens [29]. Furthermore, ASM cells obtained from *CD38* deficient mice show attenuated intracellular calcium responses to spasmogens [6]. Together, these results indicate a significant role for *CD38* in calcium signaling and contractility of ASM and AHR. Therefore, delineating the mechanisms that regulate its expression can lead to better understanding of changes in ASM function in diseases such as asthma.

MiRNAs are known to play critical roles in the regulation of gene expression and in disease pathogenesis [24]. Recent studies have identified several miRNAs whose expression in ASM cells is down-regulated by inflammatory cytokines [17,30,31]. There is also evidence for miRNA regulation of ASM contractility [31] and relaxation [32] as well as ASM phenotype [30,33]. Increased ASM mass due to growth factor-driven ASM cell hypertrophy and hyperplasia is also a feature of diseases such as asthma [34–37] and COPD [38]. Recent reports have identified the specific role of miR-221 and miR-10a in the regulation of ASM proliferation [33,39]. It is interesting to note that miR-10a targets two key signaling molecules involved in cell proliferation, i.e., PI3 kinase and ERK MAP kinase. MiR-10a suppresses the expression of the catalytic subunit of PI3 kinase, leading to decreased AKT phosphorylation.

In this study, we identified miR-708 as a regulator of *CD38* in HASM cells. We further characterized miR-708 in HASM on the basis that its expression in HASM cells is regulated by the inflammatory cytokine *rh*-TNF-α, which is elevated during allergic asthma [40,41], as well as its differential expression in cells from asthmatics versus non-asthmatics, and therefore its potential to regulate the expression of genes involved in signaling mechanisms regulating inflammation. We report that miR-708 down-regulates *CD38* expression through mechanisms that involve direct binding to the 3’UTR as well as indirectly by regulating JNK MAP kinase and PI3 kinase signaling in HASM cells. Among the numerous potential *CD38* 3’UTR binding targets that we have identified, miR-708 and miR-140-3p [17] appear to play major roles in the regulation of *CD38* expression in HASM cells. We also observed that transfection of cells with both miR-140-3p and miR-708 has no additive or synergistic effects on CD38 enzymatic activity, suggesting that either miRNA is capable of independently regulating *CD38* expression in HASM cells.

In the current study, we have shown that over-expression of miR-708 through transfection causes increased PTEN expression and an associated decrease in AKT phosphorylation. *PTEN* expression in certain cancers is regulated by miRNAs. A recent study reported that miR-221 and miR-222 inhibit *PTEN* expression by binding to the 3’UTR, while knockdown of these miRNAs causes induction of *PTEN* expression [42]. *PTEN* expression in human hepatocellular cancer cells is also under the regulation of miR-21 [43]. Knockdown of miR-21 causes increased *PTEN* expression in these cells. Regulation of *PTEN* expression in these cancer cell lines by miRNAs has a direct effect on tumor cell proliferation, migration and invasion. These studies suggest that miRNA regulation of PI3 kinase signaling in ASM cells by altering the expression of *PTEN* would have a profound impact on cell proliferation. In this context, a previous study demonstrated that in ASM cells obtained from asthmatics, the PI3 kinase/AKT pathway has a major contribution to cell proliferation [44]. However, in ASM cells obtained from non-asthmatics, cell proliferation appears to be regulated by ERK MAP kinase signaling [44]. In the current study, we found that miR-708 also regulates the phosphorylation of JNK MAP kinase in NA-HASM cells. This effect appears to be a consequence of increased expression of MKP-1, a MAP kinase phosphatase. Why this enhanced MKP-1 expression is not accompanied by decreased phosphorylation of p38 and ERK MAP kinases is not clear. However, it is very likely that the expression of some of the upstream kinases involved in the p38 and ERK MAP kinase pathways may be increased, compensating for the increased MKP-1 expression. It is worth noting that loss of *PTEN* in certain cancer cells results in increased JNK activation independent of AKT and implicating JNK as a component of the PTEN/PI3 kinase signaling cascade [45]. Thus, it is likely that the decreased JNK MAP kinase phosphorylation observed in this study may stem from increased PTEN expression. Decreased JNK activation in ASM cells has been shown to have an effect on *CD38* expression through transcription factors NF-κB and AP-1 [8]. Our study also shows that ASM cells obtained from asthmatics have increased constitutive as well as *rh*-TNF-α-induced expression of miR-708 compared to expression in cells from non-asthmatics. The functional effect of increased miR-708 expression in these cells remains to be determined. In a prior study, we reported a differential induction of *CD38* expression in response to *rh*-TNF-α treatment in ASM cells from asthmatics [20] although an enhanced half-life of CD38 transcript appears not to be an underlying mechanism for this differential induction.

## Conclusions

In summary, this study provides evidence for miR-708 regulation of *CD38* expression in human ASM cells. This regulation stems from direct 3’UTR binding to the transcript as well as through regulation of signaling involving PI3 kinase and JNK MAP kinase pathways. The inhibition of PI3 kinase signaling by miR-708 involves induction of *PTEN* expression which is likely to have a significant impact on ASM cell proliferation and AHR, the latter through inhibition of expression of *CD38* and potentially other pro-inflammatory genes. Our findings suggest the possibility that miR-708 can be used as a potential therapeutic strategy to inhibit ASM cell proliferation and contractility.

## Abbreviations

HASM: Human airway smooth muscle
AHR: Airway hyperresponsiveness
PTEN: Phosphatase and tensin homolog
UTR: Untranslated region
JNK-c: Jun N-terminal kinase
MAP kinase: Mitogen-activated protein kinase
MKP: 1-MAP kinase phosphatase 1
TNF-α: Tumor necrosis factor-α
PI3 kinase: Phosphoinositide 3-kinase
ERK: Extracellular signal-regulated kinase
